# Bisdemethoxycurcumin Attenuated Renal Injury via Activation of Keap1/Nrf2 Pathway in High-Fat Diet-Fed Mice

**DOI:** 10.3390/ijms23137395

**Published:** 2022-07-02

**Authors:** Xiaoqin Ding, Yan Chen, Lina Zhou, Ruoyun Wu, Tunyu Jian, Han Lyu, Yan Liu, Jian Chen

**Affiliations:** Institute of Botany, Jiangsu Province and Chinese Academy of Sciences, Nanjing 210014, China; dingxiao_qin@126.com (X.D.); chenyan20220526@163.com (Y.C.); linazhou1124@163.com (L.Z.); dayea999@126.com (R.W.); jiantunyu1986@163.com (T.J.); xiaohan1814@163.com (H.L.); malida66@126.com (Y.L.)

**Keywords:** bisdemethoxycurcumin, chronic kidney disease, high-fat diet, Keap1/Nrf2 pathway, oxidative stress

## Abstract

Bisdemethoxycurcumin (BDMC), a principal and active component of edible turmeric, was previously found to have beneficial effects on metabolic diseases. Chronic kidney disease (CKD) may benefit from its potential therapeutic use. Using a high-fat diet (HFD)-fed mouse model, we examined the effects of BDMC on renal injury and tried to determine how its associated mechanism works. A number of metabolic disorders are significantly improved by BDMC, including obesity, hyperglycemia, hyperinsulinemia, hyperlipidemia and inflammation. Further research on renal histopathology and function showed that BDMC could repair renal pathological changes and enhance renal function. Moreover, decreased serum malondialdehyde (MDA), elevated superoxide dismutase (SOD) activity, and the inhibition of renal reactive oxygen species (ROS) overproduction revealed the alleviation of oxidative stress after BDMC administration. In addition, renal Kelch-like ECH-associated protein 1/nuclear factor erythroid 2-related factor 2 (Keap1/Nrf2) pathway was activated in BDMC-treated mice. In conclusion, these findings demonstrated BDMC as a potential therapy for HFD-induced CKD via the activation of the Keap1/Nrf2 pathway.

## 1. Introduction

Due to the development of the economy and changes in people’s lifestyles, obesity prevalence has increased dramatically worldwide in the last few decades [1]. Metabolic syndrome is a cluster of hypertension, hyperuricemia, dyslipidemia and abnormal glucose metabolism characterized by obesity [2]. Accumulating evidence has well demonstrated that obesity may result in lipotoxicity in organs such as adipose tissue and liver. And there have been several studies initially revealing that abnormal lipid metabolism contributes to the development and progression of chronic kidney disease (CKD) [3,4], including renal structural and functional changes [5]. Although it is well known that CKD is a critical health threat, little information is available documenting the early effects of abnormal lipid metabolism in the kidney.

Tubular epithelial cell, mesangial cell, and glomerular cell apoptosis is a key factor in the development of CKD [6,7]. Several studies have proved that lipid metabolism impairment in obesity may cause renal cell apoptosis [8,9]. Oxidative stress plays an important role in the development of renal injury related to obesity [10,11]. Increased circulating free fatty acid (FFA) levels will lead to increased uptake by kidney cells, and intracellular lipid accumulation causes leading oxidative stress and inflammation [12,13]. The lipotoxic effect caused by FFA accumulation is owing to reactive oxygen species (ROS) overproduction and mitochondrial damage [14], leading to oxidative stress and increased apoptosis [7]. Hence, antioxidant defense capability is vital for sustaining cellular redox homeostasis and renal function in high-fat diet (HFD)-induced kidney injury. As a cellular defense system against oxidative stress, Kelch-like ECH-associated protein 1/nuclear factor erythroid 2-related factor 2 (Keap1/Nrf2) is thought to be critical [15]. On exposure to oxidative stress situations, the oxidation of Keap1 at specific cysteine residues inhibits the ubiquitination of Nrf2. Thus, Nrf2 can enter the nucleus to bind to antioxidant response elements, leading to the transcription of coordinated gene-encoded proteins involved in the improvement of cellular antioxidant capacity, including Heme Oxygenase-1 (HO-1), superoxide dismutase (SOD), and others [16]. It has been confirmed that renal lipid deposition is accelerated under the inhibition of Nrf2 in obesity-associated kidney damage [17], whereas Nrf2 activators effectively ameliorate oxidative stress and inflammation in animal models of kidney disease [18]. Therefore, the attenuation of oxidative stress by targeting the Keap1/Nrf2 pathway in the kidney may be a potential approach to treating obesity-related CKD.

Turmeric (*Curcuma longa*) has been frequently employed as a nutritional complement or herbal remedy for thousands of years [19,20]. Besides curcumin, which has been well investigated in the alleviation of metabolic diseases [21], bisdemethoxycurcumin (BDMC) is also a principal and active component of turmeric [22]. It has been confirmed that BDMC could efficiently protect against renal fibrosis [23], and dietary BDMC suppressed lipid accumulation in adipocytes and lowered body weight gain in HFD-fed mice [24]. However, the improvement effect of BDMC on obesity-related CKD has not been well studied. Thus, the present study is aimed to evaluate the ameliorative effects of BDMC on renal oxidative stress and renal injury in HFD-fed mice, and attempts to explore its potential mechanisms.

## 2. Results

### 2.1. BDMC Alleviated Metabolic Disorders in HFD-Fed Mice

**Figure 1:**
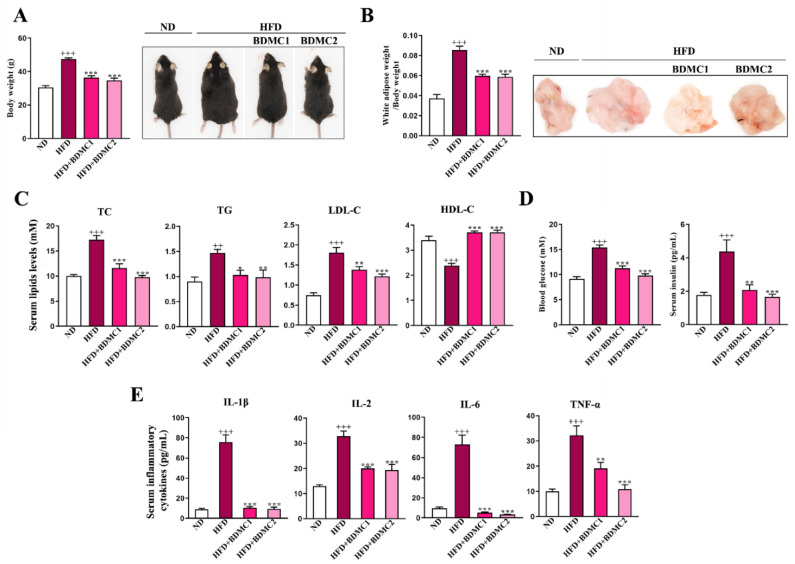
BDMC alleviated metabolic syndrome in HFD-fed mice. (**A**) Body weight and representative images of the mice. (**B**) Weight and representative images of white adipose of the mice. (**C**) Serum lipids (TC, TG, LDL-C and HDL-C) in the mice. (**D**) Fasting blood glucose and serum insulin levels of the mice. (E) Serum inflammatory cytokines (IL-1β, IL-2, IL-6 and TNF-α) levels of the mice. Bars show the mean ± SEM, N = 8. ^++^ *p* < 0.01, ^+++^ *p* < 0.001 vs. ND, and * *p* < 0.05, ** *p* < 0.01, *** *p* < 0.001 vs. HFD.

As shown in Figure 1, HFD-fed mice demonstrated elevated body weight and white adipose weight, increased blood glucose and insulin, raised serum total cholesterol (TC), triglyceride (TG) and high-density lipoprotein cholesterol (HDL-C) levels, and a decreased serum low-density lipoprotein cholesterol (LDL-C) level. What is more, HFD-fed mice exhibited increased serum inflammatory cytokines compared with Normal diet (ND)-group mice. These data showed typical metabolic syndrome in HFD-fed mice, including overweight, hyperglycemia, hyperinsulinemia, hyperlipidemia and inflammation. BDMC1 and BDMC2 treatment significantly decreased body weight, white adipose weight, serum glucose, insulin, TC, TG, and HDL-C levels while elevated serum LDL-C level, as well as decreased serum inflammatory cytokines in mice fed with HFD (Figure 1). These data suggested that BDMC significantly alleviated multiple metabolic disorders induced by HFD in mice.

### 2.2. BDMC Protected against HFD-Induced Renal Damage

**Figure 2:**
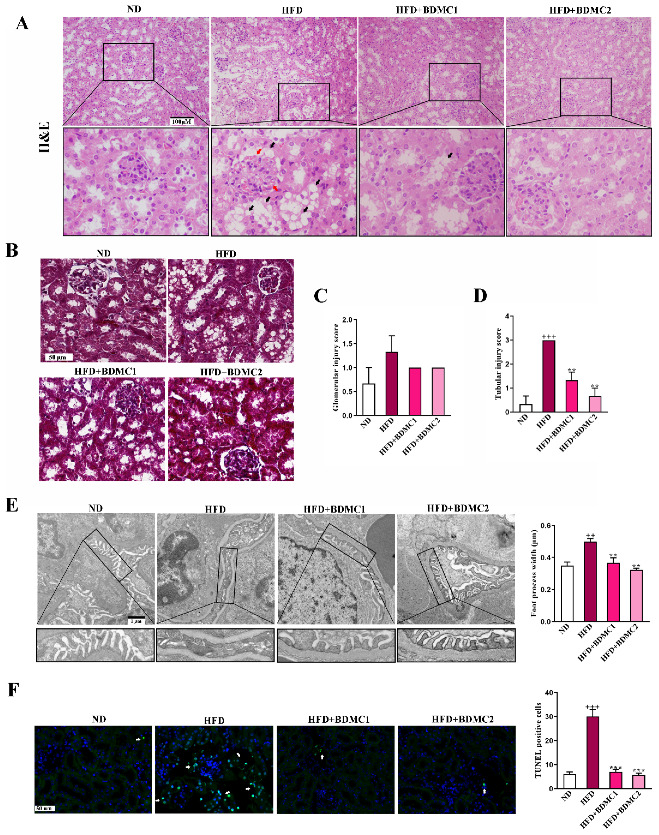
BDMC alleviated histopathological changes in the kidney of HFD-fed mice. (**A**) Renal H&E staining of the mice. BDMC reduced glomerular mesangial expansion and vasodilation (red arrow) and tubular vacuolar changes (black arrow) in the kidney of HFD-fed mice (scale bar: 100 μM, nether: higher magnification). (**B**) Renal Masson staining of the mice (scale bar: 100 μM). (**C**,**D**) Glomerular injury and tubular injury score of the mice. (**E**) TEM analysis in the kidney and the measurement of mean foot process width showed the alleviation of glomerular foot process effacement by BDMC in HFD-fed mice (scale bar: 1 μM, nether: higher magnification). (**F**) Renal Tunel staining of the mice (white arrow: apoptosis cells, scale bar: 50 μM). Bars show the mean ± SEM, N = 3. ^++^ *p* < 0.01, ^+++^ *p* < 0.001 vs. ND, and ** *p* < 0.01, *** *p* < 0.001 vs. HFD.

**Figure 3:**
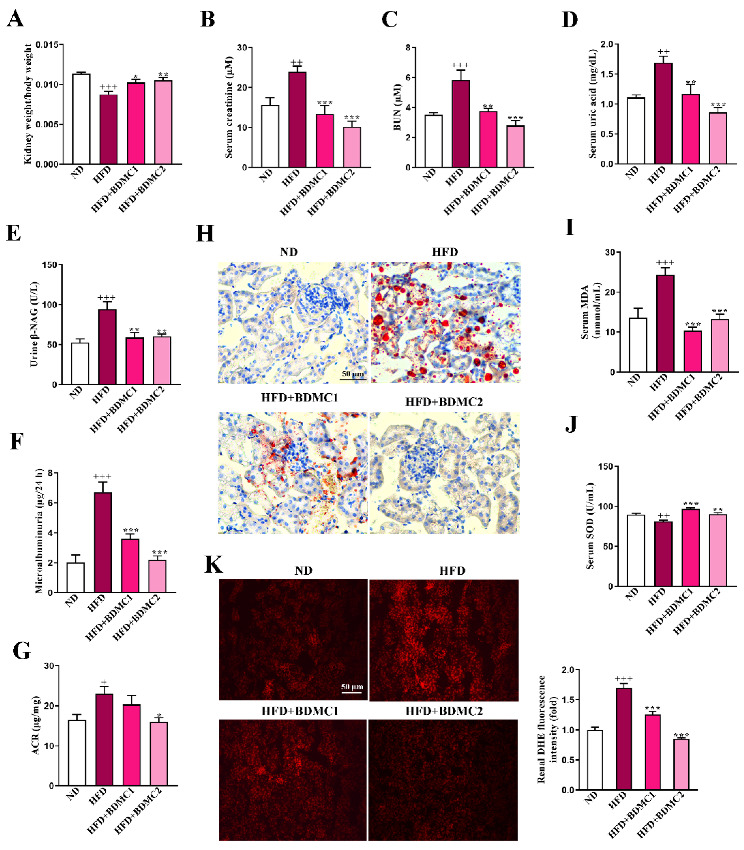
BDMC improved kidney function, reduced renal lipid accumulation and protected systematic and renal oxidative stress of HFD-fed mice (N = 8). (**A**) kidney weight index (N = 8), (**B**–**D**) serum creatinine, BUN and serum uric acid (N = 8), (**E**–**G**) uric β-NAG, microalbuminuria and ACR (N = 8), (**H**) Renal Oil red O staining, (**I**,**J**) serum MDA and SOD activity levels of HFD-fed mice (N = 8). (**K**) Renal DHE staining (magnification × 200) and the fluorescence intensity analysis of the mice (N = 4). Bars show the mean ± SEM. ^+^ *p* < 0.05, ^++^ *p* < 0.01, ^+++^*p* < 0.001 vs. ND, and * *p* < 0.05, ** *p* < 0.01, *** *p* < 0.001 vs. HFD.

To observe the pathological changes in the kidney of HFD, renal sections were stained with hematoxylin and eosin (HE), Tunel and Masson. The HFD group showed mesangial expansion and vasodilation in the glomeruli (red arrow) and macrovesicular fatty changes in the tubule (black arrow). While there was no obvious collagen deposition as shown in the renal Masson staining of the HFD-fed mice. HFD-fed mice presented relatively elevated glomerular injury score and more significant elevated tubular injury score. BDMC treatment ameliorated kidney lesion and significantly reduced tubular injury score, indicating that BDMC reversed kidney steatosis and delayed the progression of obesity-associated CKD (Figure 2A–D). What is more, podocyte foot process effacement and mean foot process width was increased in the HFD-fed mice, which was confirmed by TEM (Figure 2E). BDMC1 and BDMC2 treatment markedly decreased mean foot process width and alleviated foot process effacement. In addition, the HFD group showed an elevated level of apoptotic cells in the kidney, which was reversed by BDMC administration (Figure 2F). The kidney index of HFD-fed mice was significantly decreased, which was reversed by BDMC1 and BDMC2 administration (Figure 3A). Mice fed with HFD developed kidney injury determined by elevated serum creatinine, blood urea nitrogen (BUN) and uric acid, urine N-acetyl-β-D-glucosaminidase (β-NAG), and microalbumin levels, as well as increased urinary albumin to creatinine ratio (ACR) level (Figure 3B–G). However, BDMC administration decreased serum creatinine, BUN, and uric acid levels, as well as down-regulated urine β-NAG, microalbumin and ACR levels. These data indicated that BDMC reversed the structure and function damage of kidney in HFD-fed mice.

### 2.3. BDMC Protected HFD-Induced Lipid Accumulation, and Systematic and Renal Oxidative Stress

To evaluate the amelioration effect of BDMC on renal lipid accumulation, we conducted renal Oil Red O staining. HFD-fed mice showed elevated lipid deposition in comparison with ND mice, which was reversed after BDMC administration (Figure 3H). As shown in Figure 3I,J, HFD-fed mice displayed increased serum malondialdehyde (MDA) and reduced SOD activity levels in comparison with ND mice, both of which were reversed by BDMC treatment. Furthermore, Figure 3K showed renal ROS overproduction through dihydroethidium (DHE) staining, and ROS production was markedly suppressed by BDMC, implying that BDMC attenuated lipid accumulation, and oxidative stress both systemically and locally in the kidneys of HFD-fed mice.

### 2.4. BDMC Activated Renal Keap1/Nrf2 System of HFD-Fed Mice

**Figure 4:**
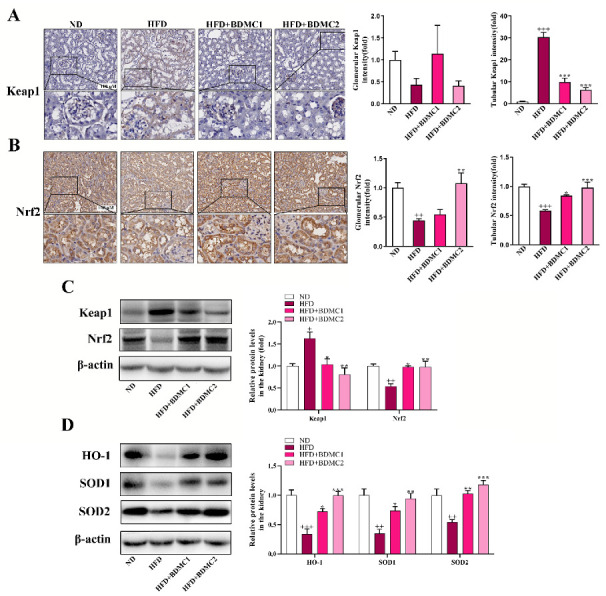
BDMC activated renal Keap1/Nrf2 system of HFD-fed mice. (**A**,**B**) Immunohistochemical and quantization of renal Keap1 and Nrf2 of the mice (scale bar: 100 μM, nether: higher magnification) (N = 4). (**C**) Western blots of renal Keap1 and Nrf2 normalized by β-actin of the mice (N = 3). (**D**) Western blots of renal HO-1, SOD1, and SOD2 normalized by β-actin of the mice (N = 3). Bars show the mean ± SEM. ^+^ *p* < 0.05, ^++^ *p* < 0.01, ^+++^ *p* < 0.001 vs. ND, and * *p* < 0.05, ** *p* < 0.01, *** *p* < 0.001 vs. HFD.

Immunohistochemistry (IHC) analysis showed that Keap1 expression in renal tubules was significantly increased under HFD conditions, whereas there was no significant change in glomeruli. Meanwhile, Nrf2 expression was significantly decreased both in renal tubules and glomeruli in HFD-fed mice. Treatment of BDMC significantly decreased Keap1 expression in renal tubules and up-regulated Nrf2 expression in renal tubules and glomeruli. Western blot analysis further confirmed the elevated renal Keap1 expression and reduced Nrf2 expression (Figure 4C) in the mice after HFD, which was reversed after BDMC administration. In addition, HFD decreased renal HO-1, SOD1, and SOD2 protein expression, which were all reversed after BDMC treatment (Figure 4D). These results demonstrated that BDMC activated the renal Keap1/Nrf2 system in HFD-fed mice.

### 2.5. BDMC Reduced Lipid Accumulation and Activated Nrf2 in HK2 Cells

**Figure 5:**
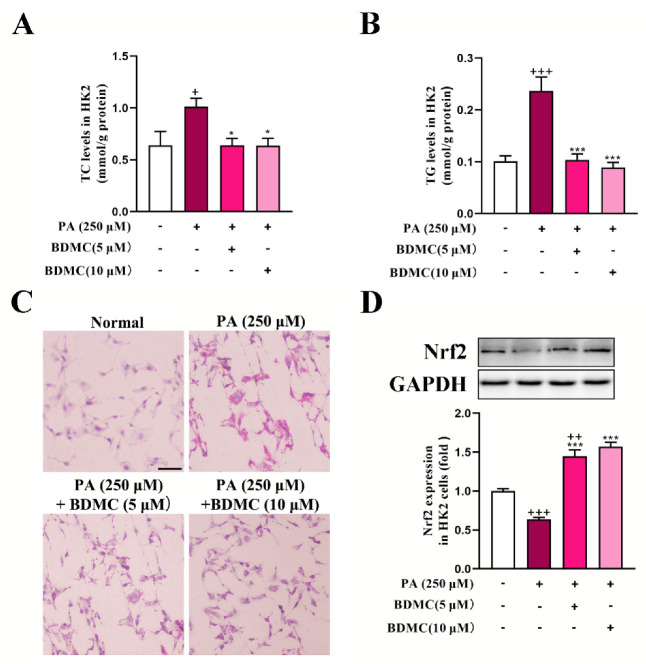
BDMC reduced lipid accumulation and activated Nrf2 in HK2 cells. (**A**,**B**) TC and TG levels in HK2 cells (N = 6). (**C**) Oil Red O staining of HK2 cells. (**D**) Western blots of Nrf2 in HK2 cells normalized by GAPDH (N = 3). Data represent the mean ± SEM. ^+^ *p* < 0.05, ^++^ *p* < 0.01, ^+++^ *p* < 0.001 vs. Normal cells. * *p <* 0.05 and *** *p <* 0.001 vs. PA-treated cells.

As shown in Figure 5A,B, PA significantly increased TC and TG levels in HK2 cells, which was reversed after BDMC administration. In addition, BDMC markedly reduced lipid deposition in PA-treated HK2 cells (Figure 5C). Furthermore, BDMC at 10 μM significantly increased Nrf2 protein expression both in normal cells and PA-treated HK2 cells (Figure 5D).

## 3. Discussion

The significant correlation between obesity and CKD has been consistently supported by epidemiological studies that explicated the increase in CKD in line with the obesity epidemic [25]. Obesity is the main feature of metabolic syndrome, a cluster of hypertension, hyperglycemia, and dyslipidemia. Accumulated studies have reported that metabolic syndrome is associated with the development of CKD [26], and that obesity itself could also threaten kidney function [4]. In this study, HFD-fed mice exhibited the major features of metabolic syndrome, including excessive weight, hyperglycemia, hyperinsulinemia, hyperlipidemia, and inflammation. Kidney pathological changes such as glomerular mesangial expansion and vasodilation, tubular vacuolar changes and podocyte foot process effacement were confirmed by renal H&E staining and TEM. In addition, kidney function impairment was observed in HFD-fed mice as demonstrated by decreased kidney index, elevated serum BUN and creatinine levels, raised uric β-NAG and microalbumin levels, as well as increased ACR levels. These data confirmed an obesity-related CKD, including renal pathological changes and renal function impairment.

BDMC is one of the three major curcuminoids (the other two are curcumin and demethoxycurcumin) isolated from turmeric, which is widely used in Asia as a dietary supplement or traditional medicine [22]. Laboratory studies and preclinical researches have demonstrated curcumin’s antioxidant and anti-inflammatory activities in metabolic diseases [19,21]. The results of human study have revealed the benefits of curcumin in patients with CKD [27]. Nevertheless, due to its instability and low water solubility, curcumin presents a low oral bioavailability, resulting in limited therapeutic applications [28]. Besides curcumin, demethoxycurcumin and BDMC have also exhibited excellent beneficial pharmacological actions, including anti-oxidant, anti-inflammatory, neuroprotective, antihypertensive, and more [29,30,31]. Compared with curcumin and demethoxycurcumin, BDMC is more stable in physiology, indicating that BDMC may have more therapeutic applications [32,33]. Several reports have indicated the uses of BDMC across anti-metabolic disorders including obesity [24] and nonalcoholic fatty liver disease [34]. Furthermore, it is reported that BDMC also has protective and preventive effects on kidney damage [23,31]. Here we identified the protection of CKD by BDMC in HFD-fed mice. In this study, HFD-induced metabolic syndrome was significantly improved by BDMC. In addition, BDMC markedly ameliorated kidney function impairment and pathological changes. These findings suggested that BDMC could reverse and relieve the progression of obesity-related CKD.

Oxidative stress and inflammation are mechanistic links between obesity and its metabolic complications [35,36]. Oxidative stress is characterized as an imbalance between the production of ROS and the antioxidant defense system. Accumulated experimental and clinical evidence suggests the central role of oxidative stress in the development of CKD [37]. Elevated ROS levels and damaged antioxidant systems are present from the early stages to the end-stage of CKD [38]. Thus, the recovery of the antioxidant system and drugs which are designed to attenuate oxidative stress are necessary and promising strategies to slow the progression of CKD. As a key transcription factor that protects against oxidative stress and regulates the inflammatory response, numerous researches have demonstrated the importance of Nrf2 in both acute and chronic kidney damage and other renal diseases [39,40,41]. Nrf2 activity is related to the intracellular concentration of Nrf2, which depends on the balance between its synthesis and degradation [15]. Nrf2 activity is repressed through Keap1, which acts as an adaptor protein in the degradation of Nrf2 under physiological conditions. An essential role of Nrf2 is to regulate the transcription of antioxidant genes, including SOD, HO-1 and more [42]. Previous studies have documented the defensive effect of Nrf2 on acute kidney injury [43,44]. Knockout of Nrf2 resulted in more severe renal impairment with graver oxidative stress in ischemia/reperfusion-associated acute kidney injury [44]. In addition, the importance of Nrf2 in the amelioration of oxidative stress, inflammation, and fibrosis in multiple-factor-related CKD—including diabetic nephropathy, membranous nephropathy and more—has been well documented [45,46,47]. Depressed renal Nrf2 expression and elevated Keap1 expression have been confirmed both in experimental DN animals and clinical CKD or DN patients [45,48,49]. While HO-1 showed different alterations relating to the severity of inflammation, comorbidities and renal damage [49]. Expression of SOD1 was lower in the kidneys of CKD patients compared with those of healthy individuals [50], and SOD2 gene expression in neutrophils from CKD patients was downregulated after LPS stimulation [51]. Moreover, IHC staining in the entire renal biopsy sample showed an overall loss of SOD3 in CKD patients [52]. A clinical trial performed to evaluate the antioxidant effects of curcumin on CKD confirmed the reduction in oxidative stress in patients with nondiabetic or diabetic Proteinuric CKD [27]. In subtotal nephrectomy-induced CKD, curcumin could upregulate Nrf2 expression to decrease renal oxidative stress and inflammation [53]. In this present study, renal lipid accumulation, extended renal Keap1 expression and depressed intracellular Nrf2 expression were observed in HFD-induced CKD mice. BDMC treatment remarkably restored renal Keap1/Nrf2-pathway inactivation and alleviated renal lipid accumulation and oxidative stress. Significant inflammation was observed in the serum but not in the kidney. Whether obesity affects renal inflammation and the effect of BDMC on renal inflammation requires further investigation. Moreover, BDMC significantly reduced lipid accumulation in HK2 cells, and activated Nrf2 in both normal HK2 cells and PA-treated HK2 cells. It has been reported that gluconeogenesis and lipogenesis were repressed in genetic Nrf2 pathway activation mice, resulting in lowering fasting glucose and insulin levels and developing less liver steatosis compared with the wild-type [54]. In addition, curcumin and dimethoxycurcumin could activate Nrf2 and up-regulate HO-1 expression in normal HepG2 cells and RAW264.7 macrophages [55,56]. Curcumin at a dose of 200 mg/kg/d could also significantly increase Nrf2 mRNA expression and partly elevate Nrf2 protein expression in the liver of normal mice [57]. Combining the existing experimental results, we tentatively inferred that curcumin might have a direct regulatory effect on Nrf2 in kidney and its regulation of lipid metabolism was also involved in the upregulation of Nrf2. Nevertheless, the direct regulatory effect of BDMC on Nrf2 still requires further experiments to confirm this hypothesis.

## 4. Materials and Methods

### 4.1. Animal Administration

Healthy seven-week-old male C57BL/6J mice were obtained from Junke Biological CO., LTD, Jiangsu, China, and housed at 25 ± 1 °C and 60–75% relative humidity, with a 12 h light/dark cycle and free access to food and water. All animal experiments were strictly performed in accordance with the guidelines of the Care and Use of Laboratory Animals (SYXK2016-0011) approved by the Animal Ethics Committee of China Pharmaceutical University. After acclimatization, 8 mice received normal diet as ND group, and other mice (N = 24) were fed with HFD diet as referred to in our previous study [58] to build the hyperlipidemic mice model. At week 10, HFD-fed mice were randomly divided into 3 groups: (1) HFD group were orally administered equal volumes of saline (N = 8); (2) BDMC1 group were orally administered BDMC (20 mg/kg/d, N = 8); and (3) BDMC2 group were orally administered BDMC (40 mg/kg/d, N = 8). BDMC (CAS: 24939-16-0, HPLC >98.0%) was obtained from Shanghai Aladdin Reagent Company, and was suspended in 0.5% sodium carboxymethyl cellulose (CMC-Na) at 2 and 4 mg/mL for oral gavage (0.1 mL/10 g body weight). 0.5% CMC-Na was used as vehicle medium to provide the same oral gavage background in mice. The original ND group was still fed the normal diet throughout the experiment, whereas other mice continued to receive a high-fat diet. Mice were placed in metabolic cages to collect their urine for 24 h throughout the final week. At week 18, mice were fasting overnight and anesthetized by sodium pentobarbital (50 mg/kg, i.*p*.), and blood samples were collected from the abdominal aorta, following the separation of perinephric, mesenteric, and inguinal white adipose and weighing. Blood was collected and centrifuged at 10,000 rpm for 10 min to obtain the serum. The kidney samples were gathered and cut into small pieces and stored in 10% formalin. The serum and remaining kidney tissues were stored at −80 °C for future use.

### 4.2. Measurement of Biomarkers in Serum and Urine

The levels of glucose, TC, TG, LDL-C, HDL-C, creatinine, uric acid and BUN in serum, and β-NAG in urine were determined with commercial assay kits (Jiancheng, Jiangsu, China). The levels of serum insulin, interleukin (IL)-2, IL-6, IL-1β, and tumor necrosis factor (TNF)-α were determined using Elisa kits (MultiSciences, Zhejiang, China). The urinary microalbuminuria level was measured using a commercial kit (Elabscience, Wuhan, China). Serum MDA and SOD activity levels were measured by commercial kits (Beyotime, Jiangsu, China).

### 4.3. Histological Analysis

Kidney tissues were fixed in 10% formalin following paraffin embedding. Then, 4 μm sections were made, and HE and Masson staining were performed as previously described [9,58]. For HE staining, sections were incubated with haematoxylin, then washed and stained with eosin. For Masson staining, sections were stained with haematoxylin, dyed with Ponceau red liquid dye acid complex and phosphomolybdic acid, and stained with aniline blue liquid and acetic acid. Afterward, renal histopathologic modifications were observed under a microscope (Olympus BX43F, Tokyo, Japan), and the glomerular injury score and tubular injury score were calculated semi-quantitatively and blindly: 0 points indicated no lesion, 1 point indicated below 25%, 2 points indicated between 25% and 50%, 3 points indicated between 50% and 75%, and 4 points indicated above 75%. Glomerular injury score was based on mesangial matrix expansion, mesangiolysis, mesangial proliferation and glomerular atrophy. Tubular injury was based on tubular dilatation, loss of the brush border and vacuolar changes [59,60].

### 4.4. Cell Culture and Treatment

The human proximal tubular cell line (HK-2) was obtained from FuHeng Cell Center, Shanghai, China. Cells were cultured in DMEM containing 10% FBS and 1% antibiotics (Invitrogen-Gibco, NY, USA) at 37 °C in 5% CO_2_. Palmitic acid PA (Macklin, Shanghai, China) was used to treat HK2 cells. For the analysis of TC and TG levels and Oil Red O staining, cells were pretreated with PA (250 μM) for 24 h, following treatment with PA (250 μM) and BDMC1 (5 μM) or BDMC2 (10 μM) for 24 h. For the analysis of Nrf2 expression, cells were pretreated with PA (250 μM) for 24 h, following treatment with PA (250 μM) and BDMC (10 μM) for 24 h, and normal cells were treated with BDMC (10 μM) for 24 h. The cell lysate was collected for the following analysis.

### 4.5. Oil Red O Staining

To measure the deposition of lipids, kidney sections and HK2 cells were stained with Oil Red O. The renal sections were fixed with 4% paraformaldehyde at 4 °C for 15 min and then rinsed with PBS three times, and HK2 cells were washed with PBS. The sections and HK2 cells were stained in the ORO solution for 30 min, then washed with 60% isopropanol, incubated with hematoxylin for 5 min, followed by immediate washing with PBS. The image was accessed by microscopy (Olympus BX43F, Tokyo, Japan).

### 4.6. Measurement of Renal ROS

DHE staining was used to determine intracellular ROS in the kidney as previously described [58]. Firstly, 4-μm-thick cryostat sections were incubated with 10 μM DHE at 37 °C for 30 min protected from light. Observation of stained kidney sections was performed under an epifluorescence microscope (Olympus CX23, Tokyo, Japan). Quantitation of DHE intensity was conducted using Image J gel analysis software. The image was split into channels via RGB splitting, and the red channel was used for analysis. Images were thresholded to separate positively stained areas, and the mean fluorescent intensity and the average area was recorded.

### 4.7. Transmission Electron Microscopy (TEM)

Small pieces of kidney tissue were swiftly cut and fixed with 2% glutaraldehyde at 4 °C and dehydrated at room temperature. After being washed by phosphate buffer, tissues were dehydrated in an alcohol gradient, then permeated and embedded in acetone. Selected fragments were cut into ultrathin sections (60–80 nm) and stained using lead citrate. Finally, the samples were viewed with a transmission electron microscope (HITACHI, Japan) at × 8000. The mean foot process width was calculated using the following formula: FPW = (π/4) × (Sigma glomerular basement membrane length/Sigma number of foot process) with Image J gel analysis software [61].

### 4.8. Cell Apoptosis Measurement

A one-step Tunel apoptosis assay kit (Beyotime, Jiangsu, China) was used to assess the apoptosis status of kidney paraffin sections. The paraffin sections were incubated in immunostaining permeabilization solution, and then washed with PBS. Tunel working solution was added and sections were incubated in a humidified chamber at 37 °C for 30 min protected from light. After incubation, the renal sections were washed three times with PBS, the digital images were captured by a confocal laser scanning microscope (Zeiss LSM 900 META, Jena, Germany), and Tunel positive cells were counted and statistically analyzed.

### 4.9. IHC Staining

For IHC, 3 μm thick paraffin-embedded renal sections were deparaffinized, hydrated and blocked, following incubation with primary antibodies including rabbit anti-Nrf2 antibody (catalog number: 16396-1-AP, Proteintech, Chicago, IL, USA) and anti-Keap1 antibody (catalog number: sc-365626, Santa Cruz, Dallas, TX, USA) overnight at 4 °C. Following diamino-benzidine (DAB) reaction, the sections were then incubated in horseradish peroxidase (HRP)-conjugated secondary antibody (Cell Signaling Technology, Danvers, MA, USA) for 30 min. Sections were photographed under a light microscope (Olympus CX23, Tokyo, Japan) and quantified with image-pro plus 6.0 software (Media Cybernetics, Rockville, MD, USA) as previously reported [62]. After intensity calibration, positive regions were extracted and separated. The positive color segmentation threshold was based on the fixed threshold value of hue, saturation, and intensity (HSI). Images segmentation and area measurement was based on the same HSI profile for all images. Integral optical density (IOD) and the area were measured and the protein expression levels were analyzed by calculating IOD/Area. Four fields of view were randomly selected from each sample.

### 4.10. Western Blot

The renal tissues were homogenized in RIPA buffer with a tissue homogenizer. The homogenate was centrifuged at 12,000 rpm for 15 min. HK2 cells were lysed in RIPA Lysis Buffer. Equal amounts of extracted proteins were separated on 10% SDS-PAGE and transferred to a PVDF membrane, blocked with blocking solution for 2 h at room temperature, and probed with the appropriate primary antibodies including anti-Nrf2 antibody (catalog number: #12721, Cell Signaling Technology, Danvers, MA, USA), anti-keap1(catalog number: sc-365626), anti SOD1(catalog number: sc-101523), anti-GAPDH (catalog number: sc- 47724), HO-1 (catalog number: sc-136256) antibodies (Santa Cruz, Dallas, TX, USA), anti-SOD2 (catalog number: 24127-1-AP), and β-actin (catalog number:66009-1) antibodies (Proteintech, Chicago, IL, USA) at 4 °C overnight. This was followed by incubation with horseradish peroxidase (HRP)-conjugated secondary antibody (Cell Signaling Technology, Danvers, MA, USA) for 1 h. Immunoreactive bands were visualized via the enhanced chemiluminescence and quantified by scanning densitometry with Image J gel analysis software. All experiments were performed in triplicate.

### 4.11. Statistical Analysis

All results were expressed as the mean ± standard error of the mean (SEM). Statistical analysis of data was performed by Graphpad software, using the one-way ANOVA with Dunnett’s post hoc test (San Diego, CA, USA). Difference was considered significant at *p* < 0.05.

## 5. Conclusions

In summary, this study revealed the beneficial effect of BDMC on HFD-induced CKD by restoration of the Keap1/Nrf2 pathway to repair renal pathological changes and enhance renal function.

## Data Availability

Data are available on request from the authors.
